# It is time to move forward into the era of *Theranostics*

**DOI:** 10.1186/s13550-018-0364-x

**Published:** 2018-01-30

**Authors:** Hojjat Ahmadzadehfar, Markus Essler

**Affiliations:** Department of Nuclear Medicine, University Hospital Bonn, Sigmund-Freud-Str. 25, 53127 Bonn, Germany

**Keywords:** Theranostics, Radionuclide therapy, Oncology, Fellowship

## Abstract

Radionuclide therapy, which until 15 years ago included only a few approved therapies, is gaining importance in the treatment of various malignancies. The future of oncology will not be limited to surgery, chemo-, antibody therapies or external radiation; it will include targeted therapy with radionuclides, which will become the standard of care for a variety of malignant diseases in combination or as an alternative to other therapies. Therefore there is a need to train Nuclear Oncologists, who are able to approach oncological diseases, promote development of radiopharmacy, understand the biology of radionuclide treatment, apply radionuclide treatments and be able to use molecular imaging such as PET/CT and SPECT/CT for treatment planning and dosimetry.

## Dear Editor,

It is important to keep in mind that our specialty (Nuclear Medicine) is not confined to diagnostic imaging and, as a matter of fact, never was in the past. We are living in exciting times featuring revolutionary development of radionuclide therapy as well as tumour imaging.

Radionuclide therapy, which until 15 years ago included only a few approved therapies, is gaining importance in the treatment of various malignancies. The future of oncology will not be limited to surgery, chemo- and antibody therapies or external radiation; it will include targeted therapy with radionuclides, which will become the standard of care for a variety of malignant diseases in combination or as an alternative to other therapies.

Currently, in many Nuclear Medicine centres in addition to radioiodine therapy for thyroid cancer, radionuclide therapies such as radioembolisation of liver tumours and radiopeptide- and radioimmunotherapies for neuroendocrine tumours and lymphomas, as well as treatment of painful bone metastases with different radionuclides, are being performed [1–5]. Most importantly, radioligand therapy for metastatic prostate cancer with Lu-177-PSMA, which has been established in some centres, shows remarkable treatment response and prolongation of overall survival in patients who do not have any other treatment options [6–10]; this further demonstrates the increasing importance of our specialty. Treatment of metastatic melanoma with melanin-binding benzamides, radioimmunotherapy for pancreatic cancer, antibody therapy for colorectal cancer and radionuclide therapy for leukaemia will be routinely offered therapies in the near future in combination with other therapies [11–14]. The chemokine ligand CXCR4 has been labelled with 177-Lu and successfully used in the treatment of multiple myeloma patients with widespread refractory disease [15].

The rapid development of radiochemistry and radiopharmacy along with breakthroughs in immunology and cell biology highlights the future importance of Nuclear Medicine. Parallel to the advancement of radionuclide therapies, we have been experiencing revolutionary growth in diagnostic imaging agents with new tracers for brain imaging as well as tumour imaging. These advances have made our specialty grow into a highly complex and unique field clearly separated from diagnostic radiology and radiation oncology.

It is time to move forward into a new era, a time of theranostics. It is time to move forward to plan and develop a new subspecialty for Nuclear Medicine physicians called “Nuclear Oncology” within the next 10 years. These specialists should be able to deal with cancer patients, approach oncological diseases, promote the development of radiopharmacy, understand the biology of radionuclide treatment, apply radionuclide treatments and be able to use molecular imaging such as PET/CT and SPECT/CT for treatment planning and dosimetry. It is mandatory for such specialists to be familiar with different oncologic therapies such as chemotherapy or immune therapy for better collaboration with all other specialties dealing with the treatment of cancer patients, including surgery, oncology, urology, gynaecology, paediatrics and pathology.

We propose the establishment of a curriculum for the training of nuclear medicine specialists to become a certified *nuclear oncologist*. The training should include a program of 1 year with rotations into specialised therapy units, PET/CT-imaging and radiopharmacy as well as outpatient care in oncology, radiation oncology and palliative medicine. During this entire period, collaboration with other specialties in oncology and participation in interdisciplinary tumour boards at a comprehensive cancer centre will be required. Apart from Nuclear Medicine learning resources, specific chapters of a medical oncology textbook, such as *Cancer* by DeVita et al. [16], should be defined as learning books for the final board certification exam taken by the candidates. In our opinion, *Nuclear Oncologists* will be an elite force to further develop our specialty and to improve patient care at highly specialised centres.

## Abbreviations

CT: computed tomography
PET: positron emission tomography
PSMA: Prostate specific membrane antigen
SPECT: single-photon emission computed tomography
